# Impact of unanswered questions on examinees’ latent traits: An item response theory perspective

**DOI:** 10.4102/ajopa.v6i0.161

**Published:** 2024-12-16

**Authors:** Joseph T. Akinboboye, Musa A. Ayanwale, David A. Adewuni, Yohanna I. Vincent

**Affiliations:** 1 Department of Science Education, Faculty of Education, Federal University of Lafia, Nasarawa, Nigeria; 2 Department of Science and Technology Education, Faculty of Education, University of Johannesburg, Johannesburg, South Africa; 3 Department of Educational Foundations, Faculty of Education, Federal University of Lokoja, Kogi, Nigeria; 4 Department of Educational Foundations, Faculty of Education, Federal University of Kashere, Gombe, Nigeria

**Keywords:** omitted responses, item response theory, Mathematics Achievement Tests, examinee characteristics, educational measurement

## Abstract

In grading examinees’ responses to test items, it is not uncommon to find that some examinees omit responses to specific items. The number of omitted responses must be considered in the psychometric analysis of test data. Omitted responses cannot be ignored, as mishandling them can jeopardise the validity of the test. This study investigates the impact of omitted responses on examinee characteristics in Mathematics Achievement Test (MAT), using item response theory (IRT) in Osun State, Nigeria. A descriptive survey research design was employed, with a sample of 600 senior secondary school 3 (SSS 3) students from eight randomly selected schools. The instrument used was a 40-item multiple-choice MAT, adapted from the West African Examinations Council’s items, with a reliability coefficient of 0.88. The instrument was content-validated by experts in Mathematics using the Lawshe content validity ratio, giving a 0.82 content validity index. The results indicate significant differences in estimated ability levels among groups, with varying probabilities of examinees producing omitted responses. The study recommends the consideration of omitted responses in IRT-based ability estimation and emphasises the importance of comparable ability groupings. This research contributes to the understanding of the complexities of educational measurement and highlights the need for careful handling of omitted responses to ensure the validity of test inferences.

**Contribution:**

This study contributes by highlighting the importance of considering omitted responses in MAT, emphasising their impact on estimated ability levels and the validity of test inferences, thus informing fairer assessment practices and enhancing the reliability of educational measurements.

## Introduction

Educational assessments are crucial in evaluating individuals’ cognitive abilities and educational progress, providing valuable insights into their academic achievements and learning trajectories (Omer, 2020). However, one common challenge in analysing examinee responses is when responses are omitted – meaning that examinees choose not to answer certain items (Dai, 2021). Omitted responses can complicate the estimation of examinee characteristics, potentially impacting the reliability and validity of assessments (Rose et al., 2017; Yuan et al., 2018). Item response theory (IRT) provides a robust statistical framework for analysing the relationship between examinee characteristics and item responses, giving insights into how missing responses might affect the accuracy of assessments (Martin et al., 2019). Item response theory models assume that the probability of a correct or incorrect response depends on the examinee’s ability and the item’s characteristics (Ayanwale & Adeleke, 2020; Dai, 2021; De Ayala, 2009).

However, when examinees omit responses, it challenges the traditional assumptions of IRT and requires careful consideration in psychometric analyses (Hong, 2021). The impact of omitted responses on examinee characteristics has been widely studied in the context of large-scale assessments, such as the Programme for International Student Assessment (PISA) and the National Assessment of Educational Progress (NAEP) (Hong, 2021; OECD, 2013; Sachse et al., 2019). These assessments often report varying rates of omitted responses across different domains and countries, emphasising the need to address this issue in psychometric analyses (Martin et al., 2019). In high-stakes tests like the West African Senior School Certificate Examination (WASSCE), examinees may choose to withdraw from test-taking, resulting in the omission of specific items (Martin et al., 2019). The prevalence of omitted responses in these assessments highlights the importance of considering their impact on test scores’ validity and reliability (Pohl et al., 2014).

Recent studies have identified three common types of missing responses: not-reached responses, missing by design and omitted responses (Dai, 2021). Not-reached responses happen when examinees do not attempt certain items because of time constraints, while missing by design refers to items intentionally omitted from the test booklet design (Braun & Von Davier, 2017). Omitted responses occur when examinees skip items deliberately or accidentally (Dai, 2021). Research in psychometrics has emphasised the importance of addressing missing responses in educational assessments to ensure the validity and reliability of test scores. Pohl et al. (2020) highlighted the need to handle missing responses carefully, as they can impact the validity of test results and bias regression coefficients when predicting competence scores from explanatory variables. Additionally, Robitzsch (2021) argued against treating omitted responses as incorrect and advocated for a more nuanced approach that considers the circumstances under which items are omitted. Studies have also examined the impact of omitted responses on the estimation of examinee abilities in IRT models. Dai (2021) investigated the influence of omitted responses on the Mantel Haenszel method in detecting differential item functioning (DIF), highlighting the potential implications for test fairness and validity. Similarly, Martin et al. (2019) explored the influence of booklet design on item response patterns, emphasising the need to consider omitted responses in large-scale assessments. In the context of Mathematics Achievement Test (MAT), the influence of omitted responses on the estimation of the examinee’s abilities is particularly significant. Kohler et al. (2017) demonstrated how omitted responses can affect the calibration of item parameters and distort the interpretation of ability scores in mathematics assessments. Moreover, Pohl et al. (2020) underscored the importance of considering missing responses in psychometric analyses of mathematics tests and highlighted their potential impact on test validity. Despite the extensive literature on omitted responses, there remains a gap in understanding their influence on examinee characteristics, particularly in the context of MATs in Nigeria. This study addresses this gap by examining the effect of omitted responses on students’ ability estimation in Osun State, Nigeria, using IRT.

### Underlying causes of missing data

In addition to examining the impact of omitted responses, it is essential to understand the underlying causes of missing data in educational assessments. Missing responses can result from various factors, including test-taking strategies, lack of knowledge, time constraints and even psychological factors such as test anxiety (Rose et al., 2017; Yuan et al., 2018). Identifying these causes is crucial for developing effective strategies to handle missing data and mitigate their impact on test validity and reliability. One common cause of missing data is nonresponse because of test-taking behaviours such as guessing, skipping items or intentionally omitting responses (Ayanwale, 2022; Oguntimehin et al., 2023; Von Davier & Sinharay, 2013). Test-takers may strategically choose not to answer certain items, affecting the completeness of the data and potentially biasing the results. Moreover, missing data can result from administrative errors, such as data entry mistakes or incomplete record-keeping (Gelman & Hill, 2006). In large-scale educational assessments, data collection processes involving multiple stakeholders and complex logistics increase the likelihood of errors and missing information. Furthermore, missing data may occur because of participant characteristics, such as language barriers, cognitive limitations or motivational factors (Enders, 2010). Students with limited English proficiency or special educational needs may struggle to understand test instructions or fully engage in the assessment process, leading to missing or incomplete responses. Little and Robbin (2014) identified three types of missing responses: ‘missing at random’ (MAR), ‘missing completely at random’ (MCAR) and ‘not missing at random’ (NMAR). Missing at random describes a specific condition related to missing data. In the context of IRT, MAR means that the probability of a response being missing is unrelated to the unobserved values (e.g. latent traits or abilities) after accounting for observed information (Enders, 2010; Hong, 2021). In simpler terms, if responses are MAR, it implies that the likelihood of an item being omitted is not systematically related to the individual’s ability. Instead, other observed variables in the analysis can explain any patterns in the missing responses (Mislevy, 2017; Hong, 2021).

For example, in an educational test scenario, if students with higher mathematical abilities are just as likely to skip an item as students with lower abilities, the missing responses would be considered MAR. When dealing with missing data assumed to be MAR in IRT models, it allows for a more straightforward analysis. It typically does not introduce significant bias into the parameter estimates. However, it is crucial for researchers to carefully assess the missing data mechanisms to make appropriate modelling decisions. Not missing at random in the context of IRT refers to a situation where the probability of a response being missing is related to the unobserved values (e.g. latent traits or abilities), even after considering the observed information (Hong, 2021). In other words, the missingness is systematic and dependent on the unobserved characteristics. For example, in an educational testing scenario, if students who perform poorly on specific questions are likelier to skip those questions, the missing data would be considered NMAR (Hong, 2021; Tabachnick & Fidell, 2007). This implies a relationship between the missing responses and the underlying abilities that extends beyond what can be explained by the observed variables. Handling NMAR data in IRT analysis can be more challenging than handling MAR data. It may require specialised models or techniques that explicitly account for the reasons behind the nonrandom missingness to ensure that the estimates of latent traits or abilities are not biased. Addressing NMAR situations is crucial in obtaining accurate and unbiased results from IRT models, as failure to account for systematic patterns in missing data can lead to distorted parameter estimates and flawed conclusions about individuals’ abilities. Missing completely at random is a term used in statistics, including IRT, to describe a specific type of missing data mechanism (Hong, 2021; Mislevy, 2017). If data are MCAR, the probability of missing values is unrelated to observed and unobserved values. In the context of IRT, MCAR implies that the likelihood of a response being missing is not systematically related to the individual’s latent trait or ability, nor is it related to any observed variables in the analysis. The missingness occurs entirely by chance. If the probability of a student omitting an item is unrelated to their actual ability and not influenced by any observed variables (such as gender, age, etc.), the missing data can be considered MCAR. The critical characteristic of MCAR is that the missingness is random and not associated with any underlying patterns or factors. Dealing with MCAR missing data in IRT analysis is relatively straightforward, as standard IRT models can be applied without introducing significant bias into the parameter estimates, assuming other modelling assumptions are met.

### Methods for handling missing data in educational assessments

Various methods and techniques have been proposed by researchers and practitioners to address missing data in educational assessments. One common approach is the use of imputation techniques, which estimate missing values based on observed data patterns (Enders, 2010). Mean imputation, regression imputation and multiple imputation are the widely used methods to handle missing responses in educational assessments (Sperrin & Martin, 2020; Vidotto et al., 2018). Another approach involves modern statistical techniques like full information maximum likelihood (FIML) estimation. Full information maximum likelihood estimation can accommodate missing data in structural equation modelling and latent variable analyses (Aitkin, 2016; Enders & Bandalos, 2001). By utilising all available data without imputing missing values, FIML estimation preserves data integrity and improves the accuracy of parameter estimates (Graham et al., 2007). In addition, maximum likelihood estimation (MLE) and multiple imputation offer robust frameworks for handling missing data in complex models such as IRT models (Aitkin, 2016; Enders & Bandalos, 2001). These methods allow researchers to account for missing data mechanisms and obtain unbiased parameter estimates while incorporating uncertainty associated with missing values. Furthermore, recent advancements in psychometric research have emphasised the importance of model-based approaches for handling missing data in IRT models (Robitzsch, Kiefer, & Wu, 2019). Model-based methods such as joint modelling and pattern mixture modelling integrate the measurement model with the missing data mechanism, enabling more accurate estimation of examinee abilities and item parameters (Robitzsch & Zeileis, 2019). Sensitivity analyses and robustness checks are also essential to assess the impact of missing data on study findings and ensure the validity of conclusions (Ulitzsch et al., 2019). These involve systematically examining the effect of different missing data assumptions or imputation methods on study results to evaluate the robustness of conclusions.

Despite these advancements, challenges remain in effectively handling missing data in educational assessments. Limited computational resources and expertise may hinder the implementation of sophisticated statistical techniques, especially in resource-constrained settings. Additionally, the choice of missing data handling methods depends on the specific characteristics of the data and the research objectives, emphasising the need for careful consideration and validation of chosen methods (Graham et al., 2007). Understanding the causes of missing data and using appropriate handling methods are crucial for ensuring the validity and reliability of educational assessments. By addressing these challenges, researchers can improve the accuracy of test scores and enhance the usefulness of assessment results for educational decision-making. This study aims to provide insights into the relationship between omitted responses and examinee characteristics by analysing data from a sample of senior secondary school students. This will contribute to a better understanding of the complexities in educational measurement.

However, it is worth noting that this study did not thoroughly explore the underlying reasons for omitted responses, such as test-taking strategies or motivation, which could provide additional insights. The purpose of this research is to provide valuable insights for improving the validity and fairness of educational testing in Nigeria, specifically in test design, scoring and interpretation. The main objective of this study is to investigate how omitted responses impact the estimation of students’ abilities in a MAT using the IRT framework. This investigation is guided by three key research questions: (1) *What is the likelihood that examinees choose not to respond to certain items?* (2) *How do examinees perceive their own ability levels?* (3) *What is the discrimination index between examinees with superior and inferior abilities?* In order to test these questions, the following hypotheses were formulated: (1) Omitted responses do not significantly affect the ability estimation of examinees with inferior abilities in IRT. (2) Omitted responses do not significantly affect the ability estimation of examinees with superior abilities in IRT. (3) There is no significant relationship between the difficulty level of an item and the likelihood of examinees omitting responses.

## Methodology

### Sample of the study

The study population comprised senior secondary school 3 (SSS 3) students from private and public secondary schools in the Osogbo Local Government Area of Osun State, Nigeria. There was a total of 313 417 students, with public schools having 284 935 students and private schools having 28 542 students. These data were obtained from the Department of Planning, Research, and Statistics, Ministry of Education, Osun State. We used a multistage sampling procedure to select our respondents. The schools were categorised as private or public, and we randomly chose four schools from each category using a simple random sampling technique. Ultimately, a total of 600 SSS 3 students from these eight schools participated in the study. The number of selected respondents from each school is as follows: 15 students from School A, 75 students from School B, 14 students from School C, 95 students from School D, 170 students from School E, 69 students from School F, 60 students from School G and 102 students from School H. This sample size was chosen based on the recommendation by Camili and Shepard (1994), who cautioned that IRT-based research with a sample size of less than 500 may result in unreliable outcomes. The researchers followed the sample size table provided by Krejcie and Morgan (1970) as a guide. Ultimately, 505 students completed the study, accounting for 83.3% of the sample.

### Research design

We utilised a descriptive survey design to examine the mathematics achievement levels among SSS 3 students in Osogbo Local Government Area. This design allowed us to collect data on existing conditions, opinions and ongoing processes related to mathematics education, giving us a comprehensive understanding of the subject matter (Akinboboye et al., 2016; Best, 1978). Our study focussed primarily on the current status of mathematics achievement among SSS 3 students while also considering past events and influences that may be relevant to their academic performance.

### Instruments

The primary instrument used for data collection was the ‘Mathematics Achievement Test’ (MAT). It consisted of 40 multiple-choice items adapted from the West African Examinations Council’s (WAEC) past test items. The test covered a wide range of topics, including index, notation and functions, approximation and estimation, logarithm, sequence and series, ratio and percentage, algebraic process, set theory, plane and solid mensuration, geometry, statistics, trigonometry, bearing, elevation and depression, variation, use of graphs, and probability. Also, five mathematics experts evaluated the test’s face to determine its validity. Content validity was also considered, as it is crucial for achievement tests and refers to how well the test adequately assesses the subject matter being studied. A test blueprint or specifications table was used to ensure the test contents aligned with the course objectives. The items were compared with the Osun State Ministry of Education scheme of work for senior secondary schools and the national Mathematics curriculum, confirming their content validity. Additionally, the instrument was administered to 30 students from a school not involved in the study to establish reliability. This aligns with the recommended sample size of Sudman (1983) and Sheatsley (1983). The responses from this group yielded a reliability coefficient of 0.88.

### Procedure

Before administering the MAT, we visited the selected schools to confirm that the teachers had adequately taught the topics covered in the test. The MAT was then administered to 600 SSS 3 students, 1 day before their regular end-of-term test, over 2 weeks. During the administration, we scored student responses for each item as right, wrong or omitted, and recorded section totals to obtain overall scores. Our test administration followed standardised procedures to ensure consistency and reliability in data collection.

### Data analysis

Our data analysis began by evaluating the assumptions of the IRT model. This involved assessing the model’s unidimensionality and fit to the data (Amusa et al., 2022; Ayanwale et al., 2020; Tobih et al., 2023). We used coefficient alpha to confirm the unidimensionality, indicating a high internal consistency among the test items. To ensure compatibility with the IRT framework, we compared the observed and predicted scores using the Pearson Chi-square statistic to examine how well individual items fit the model. A larger proportion of items showed a good fit. Next, we scored the student responses for each item as right, wrong or omitted. We recorded the section totals and added them to obtain the overall score. These total scores were then converted to Z-scores to group examinees by ability. We calculated the probability of a correct response for each examinee and item, using MLE with the one-parameter (1-PL) model. The study assessed the abilities of 505 examinees using ConstructMap, an IRT estimating programme, in three different ways. In the first approach, omitted responses were considered wrong responses. In the second approach, omitted items were treated as if they were not part of the test administered to the examinee. In the third approach, omitted items were treated as if the examinee responded correctly. To compare the performances of examinees with a high probability of omission (inferior) and those with a low probability of omission (superior) in the three scenarios, we used a test of difference (analysis of variance).

### Ethical considerations

Ethical considerations were a top priority throughout our study to safeguard the rights and well-being of respondents. We secured informed consent from the schools and students involved, clearly explaining the study’s purpose and procedures. Confidentiality of responses was rigorously maintained, with respondents assured that their academic performance would not be affected by their choice to participate or not. It is important to note that participation in the study was entirely voluntary, and students could withdraw at any time without any negative consequences. Importantly, the Faculty of Education at the Federal University of Lafia in Nasarawa granted ethical clearance for the study, with ethical reference number FEEC 2-2023-051, on 16 October 2023. This clearance aligns with the guidelines outlined in the Declaration of Helsinki, which articulates ethical standards for research involving human subjects.

## Results

**FIGURE 1:**
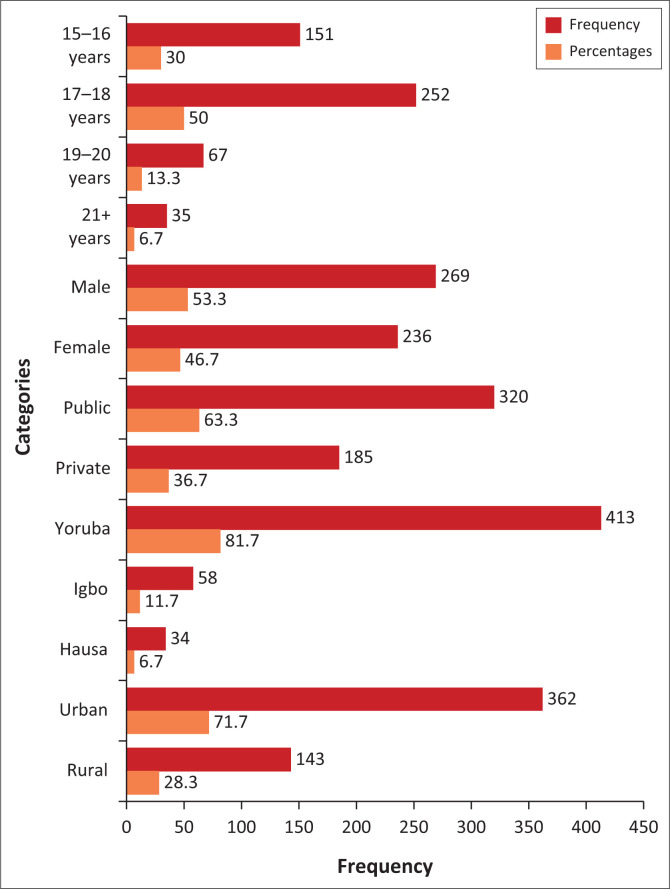
Demographic characteristics of the respondents (*n* = 505).

Figure 1 provides insight into the characteristics of the respondents in the study. The analysis reveals that most respondents are between the ages of 15 years and 18 years, representing 80% of the sample.

This indicates the importance of understanding how unanswered questions affect the latent traits of this age cohort, highlighting potential developmental differences in their responses. The gender distribution among respondents shows a slight skew towards male participants, making up 53.3% of the sample compared to 46.7% of females. This gender imbalance prompts considerations of potential gender-specific effects on manifesting latent traits in response to unanswered questions. Also, regarding school type, a larger proportion of respondents attend public schools (63.3%) compared to private institutions (36.7%). This difference emphasises the need to account for variations in educational contexts and resource availability, which could influence the prevalence and implications of unanswered questions on latent trait estimation within the IRT framework. Ethnic diversity among respondents is also noteworthy, with the majority identifying as Yoruba (81.7%), followed by Igbo (11.7%) and Hausa (6.7%). Lastly, the geographic distribution reveals that more respondents received schooling in urban areas (71.7%) compared to rural settings (28.3%). This geographic disparity emphasises potential differences in access to educational resources and support systems, which may influence the impact of unanswered questions on latent trait estimation across diverse geographic contexts.

### Research question 1

*What probability do the examinees possess in choosing not to respond to certain items?*

In order to address this research question, we calculated the probability of examinees choosing to skip (miss) an item. These probabilities were then grouped into different classes based on the likelihood of omitting items. If the probability was less than 0.1, it was considered negligible. A probability between 0.1 and 0.2 was categorised as low, while a probability greater than 0.2 but less than 0.4 was categorised as high. Once the probability exceeded 0.4, it was considered very high. The descriptive analysis revealed that 69 examinees (13.6%) had a negligible probability, 314 examinees (62.2%) had a low probability, 70 examinees (13.9%) had a high probability and 52 examinees (10.3%) had a very high probability. Overall, it can be concluded that 75.8% of the examinees (with superior ability) were likely to answer the questions, while only 24.2% of the examinees (with inferior ability) had a very high probability of choosing not to respond to some of the items.

### Research question 2

*How is the examinees’ comparison in terms of their ability levels?*

To answer the research question, the comparative ability of the examinees was computed across the participating schools and the result is shown in Table 1.

**TABLE 1:** Comparative ability of examinees across the participating schools.

Score interval	Schools								Total
	A	B	C	D	E	F	G	H	
0–10	3.000	5.000	0.000	0.000	31.000	11.000	8.000	10.000	68.000
11–20	2.000	39.000	9.000	40.000	73.000	23.000	21.000	49.000	256.000
21–40	1.000	41.000	2.000	20.000	56.000	6.000	13.000	42.000	181.000
Total	6.000	85.000	11.000	60.000	160.000	40.000	42.000	101.000	505.000
Mean score	13.400	20.410	17.460	30.080	17.520	14.880	16.210	22.590	20.140
s.d.	6.110	6.700	4.930	5.120	7.610	6.650	5.920	8.290	8.310

s.d., standard deviation.

Table 1 illustrates the similarity in the average scores of the examinees across the groups, ranging from 13.4 to 30.8. Additionally, the standard deviations are evenly distributed between 4.93 and 8.31. The proportions of examinees falling into each score interval are also similar across the groups. These findings indicate that examinees in the different groups possess comparable levels of ability.

### Research question 3

*What is the discrimination index between examinees of superior and inferior abilities?*

To address this research question, the item discrimination index for each item was calculated. The results are presented in Table 2.

**TABLE 2:** Item discrimination indices of the 40 items that made up the test.

Items	Discrimination indices
Q1	0.130
Q2	0.150
Q3	0.160
Q4	0.210
Q5	0.200
Q6	0.250
Q7	0.180
Q8	0.150
Q9	0.170
Q10	0.260
Q11	0.170
Q12	0.260
Q13	0.280
Q14	0.250
Q15	0.350
Q16	0.240
Q17	0.080
Q18	0.290
Q19	0.090
Q20	0.230
Q21	0.210
Q22	0.250
Q23	0.250
Q24	0.160
Q25	0.290
Q26	0.060
Q27	0.250
Q28	0.270
Q29	0.310
Q30	0.260
Q31	0.240
Q32	0.280
Q33	0.320
Q34	0.340
Q35	0.260
Q36	0.290
Q37	0.290
Q38	0.290
Q39	0.300
Q40	0.240

Table 2 shows the item discrimination index for each of the items. The first column presents the indices from items 1–40. The descriptive analysis for the item discrimination indices ranged from 0.060 to 0.350, and the mean discrimination index was 0.230 with a standard deviation of 0.070. In practice, values of the discrimination index will seldom exceed 0.500 because of the differing shapes of the item and total score distributions. Ayanwale et al. (2024) classified item discrimination as ‘good’ if the index is above 0.30, ‘fair’ if it is between 0.10 and 0.30, and ‘poor’ if it is below 0.10. As the mean discrimination index is between 0.2 and 0.3, we can conclude that the test discriminates at least fairly between examinees of superior and inferior ability.

**Hypothesis 1:** There is no significant impact of omitted response on the ability estimation of inferior ability examinees in IRT.

To test this hypothesis, the abilities of the 505 examinees were determined by MLE available in an IRT estimating programme, ConstructMap, which outputs the mean ability of the examinee based on each of the items. It was carried out under three conditions. The first is one in which omitted items were treated as is normally done (wrong or incorrect), the second is one in which omitted items were treated as if they were not part of the test given (not administered) to the examinee, and the third is one in which omitted items were treated as if the examinees got them right (correct). A test of difference via analysis of variance was conducted to determine if a difference exists among the three experimental conditions of ‘omitted correct’, ‘omitted missed (not administered)’ and ‘omitted wrong (incorrect)’ for inferior ability examinees as identified by the MLE statistical procedure. The result was as presented in Table 3.

**TABLE 3:** Test of difference in means of ability estimated for inferior ability examinees under the three conditions.

Sources	Sum of square	*df*	Mean square	*F*	Sig.
Between groups	7.854	2	3.927	-	-
Within groups	2.906	117	0.025	-	-

**Total**	**10.760**	**119**	**-**	**158.130**	0.000*

*df*, degree of freedom, Sig., significance.

* , *p* < 0.05.

Table 3 shows that the *F*-value obtained for inferior examinees was 158.133 at *p* = 0.000. As the *p*-value is less than 0.05, the null hypothesis cannot be accepted; hence we reject the null hypothesis and can conclude that there is a significant difference in the means of ability estimated for inferior ability examinees in the three conditions.

Table 4 shows the descriptive analysis for ability estimation of inferior ability examinees under the three conditions. The range of ability was highest for the omitted correct category (Δɵ = 1.16) followed by omitted missed (Δɵ = 0.75) and lowest for the omitted wrong condition (Δɵ = 0.65).

**Hypothesis 2:** There is no significant influence of omitted response on the ability estimation of superior ability examinees in IRT.

**TABLE 4:** Descriptive analysis for ability estimation of inferior ability examinees under the three conditions.

Treatment	*n*	Mean	s.d.	s.e.	Minimum	Maximum	Δɵ
Omitted correct	40	0.2388	0.186	0.029	−0.540	0.620	1.160
Omitted missed (not administered)	40	−0.0290	0.142	0.023	−0.320	0.430	0.750
Omitted wrong (incorrect)	40	−0.3860	0.139	0.022	−0.680	−0.030	0.650

**Total**	**120**	**−0.0590**	**0.301**	**0.028**	**−0.680**	**0.620**	**1.300**

s.d., standard deviation; s.e., standard error.

To test this hypothesis, the procedure employed in testing hypothesis 1 was also employed here and the results are shown in Table 5.

**TABLE 5:** Test of difference in means of ability estimated for superior ability examinees under the three conditions.

Sources	Sum of square	*df*	Mean square	*F*	Sig.
Between groups	8.550	2	4.280	-	-
Within groups	1.860	117	0.020	-	-

**Total**	**10.410**	**119**	**-**	**269.760**	0.000*

*df*, degree of freedom; Sig., significance.

* , *p* < 0.05.

Table 5 shows that the *F*-value obtained for superior examinees was 269.756 at *p* = 0.000. As the *p*-value is less than 0.05, the null hypothesis cannot be accepted; hence we reject the null hypothesis and can conclude that there is a significant difference in the means of ability estimated for superior ability examinees in the three conditions.

Table 6 shows the descriptive analysis for ability estimation of superior ability examinees under the three conditions. The range of ability was highest for the omitted correct category (Δɵ = 0.690) followed by omitted missed (Δɵ = 0.530) and lowest for the omitted wrong condition (Δɵ = 0.490).

**Hypothesis 3:** There is no significant relationship between the difficulty level of an item and the probability of the examinee omitting responses.

**TABLE 6:** Descriptive analysis for ability estimation of inferior ability examinees under the three conditions.

Treatment	*n*	Mean	s.d.	s.e.	Minimum	Maximum	Δɵ
Omitted correct	40	1.070	0.120	0.020	0.540	1.230	0.690
Omitted missed (not administered)	40	0.810	0.130	0.020	0.470	1.000	0.530
Omitted wrong (incorrect)	40	0.420	0.133	0.020	0.140	0.630	0.490

**Total**	**120**	**0.770**	**0.296**	**0.030**	**0.140**	**1.230**	**1.090**

s.d., standard deviation; s.e., standard error.

In order to test this hypothesis, the probabilities of the students’ omitting responses were obtained and also the difficulty of the 40 items. A Pearson correlation was conducted between the two and the result was as presented in Table 7.

**TABLE 7:** Relationship between probability of missing an item and the difficulty of the item.

Variable	Mean	s.d.	*r*	*p*
Probability of missing	0.150	0.170	0.070	0.667*
Difficulty	0.500	0.120	-	-

s.d., standard deviation.

* , *p* > 0.05.

Table 7 shows the value of Pearson to be *r* = 0.070 and *p* = 0.667. As the *p*-value is greater than 0.05, the null hypothesis cannot be rejected. Hence, we accept the null hypothesis and can then conclude that there is no significant relationship between the difficulty level of an item and the probability that an examinee will produce an omitted response.

## Discussion

The results of this study offer valuable insights into the intricate dynamics of educational measurement and assessment, particularly within the context of Mathematics achievement tests using IRT in Osun State, Nigeria. These insights hold significant implications for the improvement of test validity, reliability and fairness, thereby enriching the broader field of educational assessment.

One notable contribution of this study lies in its exploration of the impact of omitted responses on examinee characteristics and ability estimation. The findings illuminate the prevalence of omitted responses among examinees, highlighting a potential challenge in accurately assessing their abilities. This underscores the importance of addressing omitted responses in psychometric analyses to maintain the validity of test scores. By shedding light on this issue, the study prompts a re-evaluation of current testing practices and emphasises the need for strategies to minimise the occurrence of omitted responses in educational assessments (Hong, 2021).

Moreover, the study’s examination of how different treatments of omitted responses affect ability estimation for examinees with varying levels of ability provides valuable insights into the nuances of psychometric analysis. The significant differences observed in ability estimation under different treatment conditions underscore the sensitivity of ability estimates to the handling of omitted responses. This emphasises the importance of employing robust statistical methods to handle missing data effectively and ensure unbiased parameter estimates in educational measurement (Enders & Bandalos, 2001).

Furthermore, the analysis of discrimination indices adds another layer of understanding to the study’s findings. Despite the challenges posed by omitted responses, the test items demonstrated adequate discrimination between examinees of different ability levels. This reaffirms the test’s ability to differentiate effectively between examinees with varying levels of ability, thereby contributing to the overall validity of the assessment. This underscores the importance of developing test items that effectively discriminate between examinees of different ability levels, ultimately enhancing the reliability and validity of educational assessments.

Relating these findings to existing empirical evidence, previous research has also highlighted the impact of missing data on the validity and reliability of educational assessments (Enders, 2010; Von Davier & Sinharay, 2013). Studies have demonstrated the need to employ robust statistical methods, such as MLE and multiple imputation, to handle missing data effectively and ensure unbiased parameter estimates in psychometric analyses (Enders & Bandalos, 2001). The current study’s emphasis on addressing omitted responses in ability estimation aligns with these recommendations, emphasising the importance of considering missing data mechanisms in educational measurement.

In light of these findings, the study emphasises the importance of addressing omitted responses in educational assessments, particularly in the context of IRT-based ability estimation. By recognising the influence of omitted responses on test validity and providing insights into effective handling methods, the study contributes to the ongoing efforts to improve the accuracy and reliability of educational assessments. Moving forward, these insights can inform the development of more robust testing practices that better serve the needs of students and educators in Nigeria and beyond.

### Recommendations

Based on the insights gained from the study, several recommendations emerge to enhance the management of omitted responses in educational assessments. Firstly, test designers and administrators should implement strategies to reduce the occurrence of omitted responses. This may involve providing clear and concise instructions to examinees and allowing sufficient time for completing the test. By creating a conducive testing environment, examinees are more likely to fully engage with the assessment process, thus minimising the chances of omitted responses. Secondly, psychometricians and researchers should use robust statistical methodologies, such as MLE, to effectively handle omitted responses during the ability estimation process. By utilising advanced statistical techniques, educators can obtain more accurate and reliable estimates of examinees’ abilities, thereby enhancing the validity and precision of assessment outcomes. Furthermore, it is crucial for educators and policymakers to recognise the potential implications of omitted responses on test validity and the interpretation of assessment results. By acknowledging the impact of omitted responses, stakeholders can implement appropriate measures to mitigate any adverse effects on the overall validity and reliability of the assessment. This may include providing targeted support and guidance to examinees and incorporating alternative assessment strategies to capture a more comprehensive understanding of students’ capabilities.

### Limitations and future work

To further advance our understanding of the implications of omitted responses in educational assessments and address the limitations of the current study, several avenues for future research could be pursued. Firstly, future investigations could delve into the underlying reasons behind omitted responses. This could involve employing qualitative methods or surveys to explore factors such as test-taking strategies, motivation levels or test anxiety that may influence examinees’ decisions to omit responses. Understanding these factors could provide valuable insights into the psychological processes at play during test-taking and inform the development of interventions aimed at mitigating the occurrence of omitted responses. Secondly, researchers could explore the broader impact of omitted responses on various aspects of test validity, including reliability and fairness, across different subject areas and educational contexts. By examining how omitted responses affect the overall psychometric properties of tests, future studies could shed light on the generalisability of findings and help identify potential areas for improvement in test design and administration.

Thirdly, it would be beneficial to investigate the effectiveness of interventions designed to reduce the incidence of omitted responses. This could involve implementing measures such as providing additional test-taking support, offering incentives for thorough completion of test items, or modifying test designs to make them more engaging and accessible to examinees. Evaluating the efficacy of such interventions could provide valuable guidance for educators and policymakers seeking to enhance the validity and reliability of educational assessments. By exploring these avenues for further research, future studies can contribute to a deeper understanding of the impact of omitted responses on educational assessments. Moreover, insights gleaned from such research endeavours can inform the development of more effective testing practices and policies aimed at promoting fair and accurate evaluation of student learning and achievement.

## Conclusion

This study explores the impact of omitting responses on mathematics achievement tests taken by examinees in Osun State, Nigeria. Using the IRT framework, the research emphasises the importance of considering omitted responses in psychometric analyses. By doing so, the study emphasises the need to ensure precise and reliable assessment outcomes. Notably, the findings provide valuable insights into the complexities of educational measurement. Specifically, the research explains how omitted responses affect the estimation of examinees’ abilities and the identification of differences among different groups of test-takers. This study contributes to a broader understanding of assessment practices and highlights the significance of addressing omitted responses in educational evaluation. By shedding light on these dynamics, the research serves as a valuable resource for educators, policymakers and psychometricians who aim to improve the accuracy and fairness of educational assessments.
